# The Endocannabinoid Activity Remodulation for Psychosis Liability in Youth (EARLY) Study: An Open-Label Feasibility Trial of Ultramicronized-Palmitoylethanolamide Oral Supplementation in Clinical High-Risk State for Psychosis

**DOI:** 10.3390/brainsci14121230

**Published:** 2024-12-07

**Authors:** Riccardo Bortoletto, Marco Garzitto, Fabiana Piscitelli, Stefano Fornasaro, Claudia Scipioni, Orietta Sepulcri, Martina Fabris, Francesco Curcio, Matteo Balestrieri, Marco Colizzi

**Affiliations:** 1 Unit of Psychiatry and Eating Disorders, Department of Medicine (DMED), University of Udine, 33100 Udine, Italy; marco.garzitto@uniud.it (M.G.); scipioni.claudia@spes.uniud.it (C.S.); matteo.balestrieri@uniud.it (M.B.); 2 Institute of Biomolecular Chemistry, National Research Council (CNR), 80078 Pozzuoli, Italy; fpiscitelli@icb.cnr.it; 3 Department of Chemical and Pharmaceutical Sciences, University of Trieste, 34127 Trieste, Italy; sfornasaro@units.it; 4 Unit of Psychiatry and Eating Disorders, Friuli Centrale Health University Authority (ASUFC), 33100 Udine, Italy; orietta.sepulcri@asufc.sanita.fvg.it; 5 Department of Medicine (DMED), University of Udine, 33100 Udine, Italy; martina.fabris@asufc.sanita.fvg.it (M.F.); francesco.curcio@uniud.it (F.C.); 6 Institute of Clinical Pathology, Friuli Centrale Health University Authority (ASUFC), 33100 Udine, Italy; 7 Department of Psychosis Studies, Institute of Psychiatry, Psychology and Neuroscience, King’s College London, London SE5 8AF, UK

**Keywords:** schizophrenia, cannabidiol, nutraceuticals, prevention, transition-age psychiatry

## Abstract

To date, no psychotropic medication has shown to effectively halt progression to psychosis among individuals at Clinical High-Risk for psychosis (CHR), fueling the search for novel therapeutic agents. Recent evidence supports Palmitoylethanolamide (PEA) signaling as a potential psychosis biomarker, also indicating a therapeutic role for its supplementation in the treatment of psychotic disorders. Nonetheless, the effect of sustained PEA intake in CHR subjects has never been explored so far. We will assess the feasibility of enrolling 20 CHR young adults presenting with attenuated psychotic symptoms (APS) in a 12-week, open-label, investigator-initiated, proof-of-concept, single-arm trial of ultramicronized-PEA (um-PEA) 600 mg/day. Once completed the 12-week phase, participants will be proposed to enter a 24-week extension phase of the study. We will examine um-PEA ability to reduce APS and psychic distress, um-PEA safety and tolerability, and the biological basis of um-PEA effect in terms of modulation of inflammatory response, endocannabinoid (eCB) signaling, and microbiome composition. Our trial aims to address an unmet clinical need in CHR subjects, providing an initial solid basis for the development of future studies evaluating the efficacy and tolerability of PEA supplementation in this group of patients.

## 1. Introduction

The Clinical High-Risk (CHR) for psychosis state has been first described over two decades ago and represents the phase preceding the potential development of full-blown psychosis [1]. Recent meta-analyses of risk of transitioning to psychosis at 2 years among CHR individuals show rates of approximately 16–19%, while risk factors accumulation (*risk enrichment*) results in increasing rates of up to 35% at 10 years [2,3]. Compared to the outdated “prodrome” concept, the CHR paradigm provides a less deterministic understanding of schizophrenia (SCZ), thus highlighting the importance of detection, assessment, and early intervention on milder symptoms preceding psychosis onset to halt disease progression [4,5,6]. However, to date, no psychotherapeutic or psychopharmacological interventions have shown to effectively prevent progression to psychosis among CHR subjects [3,7], nor to relieve them from attenuated psychotic symptoms (APS) [8], negative symptoms [9], or impaired global functioning [10]. Compelling evidence of the endocannabinoid (eCB) system early disruption in the neurophysiological pathway leading to schizophrenia onset has fostered the search for novel medications (e.g., cannabidiol, CBD) targeting the eCB system for both psychosis patients and CHR individuals [11,12,13,14,15,16].

To this extent, growing research is focusing on the role of the eCB-like compound Palmitoylethanolamide (PEA) as a potential therapeutic agent in the context of several neuropsychiatric conditions [17,18,19]. PEA is a naturally occurring N-Acylethanolamine (AE), proven to modulate the central and peripheral neuroimmune systems and to exert protective functions against glutamate neurotoxicity [20,21,22]. It directly activates the Peroxisome Proliferator Activated Receptor-α (PPAR-α) and G protein-coupled receptor 55 (GPR55), allosterically modulates the Transient Receptor Potential Vanilloid 1 (TRPV1), and indirectly interacts with type 1- (CB1) and type 2- (CB2) cannabinoid receptors [23], sharing similar pharmacodynamic properties with the phytocannabinoid CBD [24]. We recently conducted a systematic review aimed at investigating the biobehavioral role of PEA in psychotic disorders [25]. Overall, evidence from clinical studies included in our reappraisal converges on the involvement of PEA in different psychotic phenotypes. A robust strain of research indicates an endogenous attempt of PEA to restore homodynamic balance under disease conditions in CHR individuals [26], non-affective psychosis patients [27,28,29,30], and affective psychosis patients [28,31] at different stages of illness. Specifically, PEA blood tone would increase in the initial phases of a psychotic disorder, while subsiding concomitantly to disease progression. Consistently, PEA levels in the central nervous system (CNS) were found to be elevated in the early stages of SCZ [32], before declining as the disease progresses [33]. Limited but promising evidence was also obtained from interventional studies assessing the potential of PEA therapeutic supplementation in SCZ patients [34], bipolar disorder patients with manic symptoms [35], and Parkinson’s disease patients with psychotic symptoms [36]. PEA showed a selective efficacy on negative psychotic (e.g., alogia) [34] and acute manic [35] symptoms, while not significantly ameliorating positive psychotic symptoms (e.g., delusions) [34,35,36]. To the best of our knowledge, to date there is no evidence of the effect of PEA treatment in the clinical stages preceding the onset of psychosis as well as to prevent the risk of disease progression.

We propose a feasibility study of sustained ultramicronized-PEA (um-PEA) supplementation in CHR individuals to address the lack of a first-line treatment for this group of subjects, focusing on study recruitment and patient acceptability.

## 2. Materials and Methods

### 2.1. Trial Design

This is a 12-week, open-label, investigator-initiated, proof-of-concept, single-arm study (Phase-2 Pilot Study) assessing the effect of um-PEA 600 mg/day in CHR individuals presenting with APS [1]. Within the first 12 months from the first participant recruitment, we will assess the feasibility of enrolling a minimum of 20 CHR participants and whether at least 80% of participants have completed the 12-week follow-up. After completing the initial 12-week phase, participants will be proposed to enter a 24-week extension phase of the study to evaluate the clinical stability of the treatment. Based on clinical judgement of the improvement obtained at that point, um-PEA might be titrated to 1200 mg/day. Each participant will undergo um-PEA treatment for a maximum of 36 weeks (Table 1). Our purpose is to examine: (i) um-PEA ability to alleviate APS and anxiety symptoms in CHR patients; (ii) um-PEA safety and tolerability; (iii) the biological basis of um-PEA effect. The study was approved by the Department of Medicine (DMED) at University of Udine (Institutional Review Board: 93/2023) in June 2023 (https://osf.io/wn2kf, accessed on 5 December 2024).

### 2.2. Sample Size Calculation

The primary analysis (see below) will focus on total severity of APS as evaluated using the Comprehensive Assessment of At-Risk Mental State (CAARMS) [1], measured in three longitudinal assessments (i.e., Feasibility phase: Baseline, T1, and T2). Accepting a type-I error (α) of 5% and a type-II error (β) of 20%, we would need a sample of 8–12 participants to detect an effect of large size (partial-η2 ≥ 0.140). This value was obtained considering a moderate-to-large test-retest reliability of measurements over time (+0.500 < r ≤ +0.700) and the absence of correction for sphericity. With an intermediate sphericity correction (ε = 0.750), the required sample size would be 10–14; in the worst case (ε = 0.500), instead, 12–19 participants would be needed. We therefore propose the recruitment of 20 participants, considered coherent with a pilot study and sufficient to evaluate feasibility outcomes of clinically significant size, also considering a low drop-out rate (i.e., ≤ 20%). When large statistically significant results are obtained on total severity of APS, specific APS severities will be further investigated (i.e., considering four P1 scales). For this secondary endpoint, on average, a sample-size of 17 participants would be sufficient to detect large effects, also considering Bonferroni’s correction for multiple independent comparisons (i.e., α = 0.012). Power analyses were carried out with G*Power-3.1.9.7 [45].

### 2.3. Objectives

#### 2.3.1. Feasibility Endpoints

We will record (i) the number of subjects giving consent for participating to the trial; (ii) the proportion of participants completing the initial 12-week follow-up. Patients will be defined as compliant in the presence of a pill count greater than 50% the expected number taken. Patients who are defined as non-compliant with the medication will be coded as protocol deviators.

#### 2.3.2. Research Endpoints

We will primarily address whether um-PEA added to treatment as usual (TAU) in CHR patients improves APS. Our secondary research questions are whether um-PEA added to TAU in CHR patients improves APS to the extent that patients no longer satisfy the diagnostic criteria for the CHR state, relieves APS-associated distress, improves CHR-related anxiety symptoms, and ameliorates social and role functioning, as well as general psychopathology and wellbeing measures. Therefore, our primary endpoint will be the assessment of APS severity as measured using the CAARMS [1]. As per the secondary endpoints: (i) APS-associated distress will be measured using the CAARMS [1]; (ii) severity of CHR-related anxiety symptoms will be measured using the Hospital Anxiety and Depression Scale (HADS) [43]; (iii) the level of impaired global functioning will be measured using the Social and Role functioning Scale [44]; (iv) clinical remission, defined as no longer meeting the APS criteria [1] will be measured using the CAARMS [1]; (v) general psychopathology and wellbeing changes will be measured using the CAARMS [1].

All the study endpoints for the 12-week clinical trial will be assessed by comparing follow-up (FUP) visits and baseline. For those participants continuing in the 24-week extension phase, change (FUP visit minus baseline) in the severity of psychotic symptoms as measured using the CAARMS will be compared with the group of those willing to discontinue um-PEA and continue with TAU. Should the psychopathological condition require TAU adjustments based on clinical judgement, this will be mentioned in a specific section of the trial case report form (CRF; https://osf.io/2vayh, accessed on 5 December 2024).

#### 2.3.3. Safety Endpoints

We will evaluate if sustained um-PEA treatment is well tolerated, with minimal side-effects. Incidence of adverse effects during the study period will be measured using the UKU side-effect rating scale [42].

### 2.4. Biological Measures

Blood tests for hematology (i.e., full blood count and hemoglobin), biochemistry (i.e., urea and electrolytes, liver function test, lipid profile), sex hormones quantification [i.e., follicle-stimulating hormone (FSH), luteinizing hormone (LH)], and hypothalamic-pituitary-adrenal axis activity-related indices quantification (i.e., morning cortisol at 8 a.m.) will be carried out at the clinical research recruitment hub as per their standard procedure. Cytokines and chemokines will be analysed on available serum samples using customized multiplex immunoenzymatic assays (Ella instrument, Bio-Techne, Minneapolis, MN, USA) to characterize the inflammatory profile. Serum samples will be collected for performing “metabolic fingerprinting” analysis using Surface-Enhanced Raman Scattering (SERS) spectroscopy, a rapid, label-free, and non-destructive method revealing molecular information about the biochemical composition of biofluids by analysing shifts in SERS spectral profiles [46,47,48]. Additionally, blood and fecal samples will be collected for the measurement of eCB mediators using liquid chromatography-mass spectrometry (LC/MS) techniques [49,50], and the determination of the microbial composition in feces using Next Generation Sequencing (NGS) of 16SRNA and shotgun metagenomics methodologies [51].

### 2.5. Study Setting and Participants

#### 2.5.1. Screening and Recruitment

The study will take place at the Unit of Psychiatry and Eating Disorders of the Udine University Hospital, a university clinical research facility in Italy. Help-seeking individuals presenting at the Unit of Psychiatry and Eating Disorders upon referral from General Practitioners (GPs) or other mental healthcare professionals from Udine catchment area and identified as CHR-APS patients by the clinical team will be considered for enrolment against inclusion and exclusion criteria. They will be involved into an internal pilot, that will progress to the open-label trial. Patients expressing an interest in participating to the study will be approached by study researchers and given a patient information sheet and consent form (https://osf.io/nrc5b, accessed on 5 December 2024).

Those who agree to take part in the study will be invited for a screening visit.

#### 2.5.2. Inclusion Criteria

The following are the participants’ inclusion criteria:

- Individuals diagnosed with CHR-APS, as defined using CAARMS criteria [1];
- Aged 18–35 years;
- To be able to understand and communicate in Italian;
- To be able to give informed consent.

#### 2.5.3. Exclusion Criteria

The following are the participants’ exclusion criteria:

- Lifetime history of a psychotic or manic episode lasting 7 days or longer;
- Active suicidal ideation indicating significant current risk or history of serious suicide attempt in the opinion of the Principal Investigator (PI), as evaluated at the screening stage;
- Lifetime neurological disorders (e.g., epilepsy, except febrile convulsions) or severe intercurrent physical illness;
- Current treatment with psychotropic medication, with the exception of Selective Serotonin Reuptake Inhibitor (SSRI) stable monotherapy (at least 8 months);
- Lifetime treatment with antipsychotic medication for more than 7 days;
- Intelligence quotient (IQ) < 70;
- Female patients who are pregnant, lactating or not using an acceptable effective form contraception if they are at risk of becoming pregnant;
- Taking part in another pharmacological trial.

#### 2.5.4. Demographics

At the time of the screening visit, socio-demographic and clinical characteristics of each participant will be recorded in the trial CRFs (https://osf.io/2vayh, accessed on 5 December, 2024), including (i) age, (ii) sex, (iii) height, (iv) weight, (v) body mass index (BMI), (vi) reason for first referral, (vii) housing situation, (viii) marital status, (ix) nationality, (x) level of education, (xi) working situation, (xii) handedness, (xiii) prior contact with child & adolescent mental health service (CAMHS), (xiv) prior contact with adult mental health service (AMHS), (xv) predating hospitalizations for mental health issues, (xvi) predating contact with social services, (xvii) predating diagnosis, (xviii) age at predating diagnosis, (xix) comorbid neurodevelopmental disorders (NDDs), (xx) comorbid physical illnesses, (xxi) impairment in daily autonomies, (xxii) intellectual disability, (xxiii) brain magnetic resonance imaging (MRI), (xxiv) childhood adversities (CAs; i.e., sexual/physical/psychological/ emotional abuse, physical/emotional neglect, exploitation, peer victimization/bullying), (xxv) any CAs, (xxvi) recent stressful life events (SLEs), (xxvii) predating treatment with any psychotropic medications, (xxviii) type of predating medication, (xxix) current treatment with any psychiatric medications, (xxx) type of current medication, (xxxi) lifetime addicting substance use (any), (xxxii) current addicting substance use (any), (xxxiii) lifetime cannabis use, (xxxiv) current cannabis use, (xxxv) lifetime alcohol use, (xxxvi) current alcohol use, (xxxvii) lifetime stimulant use, (xxxviii) current stimulant use, (xxxix) NDDs family history, (xl) major mental health disorders (MMHDs) family history, (xli) substance use disorders (SUDs) family history, (xlii) suicide/suicide attempt family history, (xliii) neurological disorders family history.

Substance use information will be collected as screening measures, using dedicated interviews [37,38,39,40].

### 2.6. Intervention

Participants will initially undertake a 12-week daily treatment with oral um-PEA 600 mg in tablet form (Normast^®^). During the 24-week extension phase of the study, the trial medication will be taken from once a day up to twice a day (600–1200 mg per day), based on clinical judgment of the improvement obtained at that point, around mealtime. Being a food supplement/nutraceutical, um-PEA can be purchased at pharmacies without a medical prescription [52]. While unknown risks cannot be excluded, serious adverse events including overdose have not been documented [53,54]. Um-PEA will be obtained by a pharmaceutical company operating under good manufacturing practice conditions with appropriate certification.

The information presented on the labels for um-PEA will comply with applicable national and local regulations. Only qualified physicians clearly given this role by the PI will prescribe the study medication on the study delegation log. Only people designated by the PI will collect medication. Only um-PEA supplied for this study will be dispensed against the study specific prescription. Full accountability records will be completed including recording the batch, expiry date, people dispensing/checking the prescription, quantity and date of drug returns, empty packaging. Nothing will be destroyed without the authorization from the PI.

Um-PEA will be stored at room temperature (<25 °C) and not kept in a refrigerator, in compliance with local regulations. It will be stored in a secure area away from other treatments and clearly marked for this study.

### 2.7. Data Management and Confidentiality

Study-related information will be stored securely at the study site. All participants’ information will be stored in locked file cabinets in areas with limited access. All laboratory specimens, reports, data collection, process and administrative forms, will be identified by a coded identification (ID) number only to maintain participant confidentiality and stored separately from records containing names or other personal identifiers. Local databases will be secured with password-protected access systems. Forms, lists, logbooks, appointment books, and any other listings that link participant ID numbers to other identifying information will be stored in a separate, locked file in an area with limited access. Participants’ study information will not be released outside of the study without the written permission of the participant.

### 2.8. Statistical Methods

#### 2.8.1. Data Verification, Statistical Monitoring, and Analysis

At the study start, a structured data verification plan will be agreed and developed by the Head Statistician (M.G.) of the research team. Quality of data will be routinely checked in terms of consistency across paper- and electronic-based entry systems. Statistical monitoring will include patient severity level, eventual withdrawals, baseline data, FUP visits data, and adverse effects. The research team will approve a Statistical Analysis Plan within the early stages of the study, and before the Head Statistician summarizes any data (i.e., with univariate and repeated measures analysis, including preliminary assessment and management of any outliers and assessment of the metric qualities of measurements, such as normality and sphericity, where necessary). Proportions will be presented for each feasibility outcome. Interim analyses are not planned. The Head Statistician will both carry out and interpret statistical analysis. Our primary analysis will involve a generalized linear model with CAARMS psychotic symptom severity as the dependent measure and time as a repeated measure (possibly supplemented with analyses at the participant level, as random effects in mixed models). We are interested in whether there is a significant benefit over time with um-PEA treatment: therefore, particular interest will be placed on time as a fixed factor on the severity of outcomes. CAARMS distress and total score as well as HADS and GF ratings will be analysed in a similar manner. We will also conduct paired tests (both parametric and non-parametric) based on baseline and endpoint assessments to examine measures of CAARMS severity, CAARMS frequency/duration, and secondary outcomes. Trend and rates of adverse effects will be reported, as well as reasons for dropouts from the trial.

#### 2.8.2. Missing Data

We expect withdrawn patients to be missing at random, and very few participants withdrawing consent. All patients having at least one post-baseline outcome measure will be included in each analysis. Nonetheless, patterns of missing data will be explored to investigate any evidence against missing at random, whose impact on the analysis will be considered by introducing imputation methods (i.e., Multiple Imputation or MissForest approach, depending on the missing data type).

### 2.9. Withdrawal of Participants

In accordance with the Declaration of Helsinki [55], all participants have the right to withdraw from the study at any time, without the need to provide a reason, and without affecting their future medical care. Participants will be informed of this right prior to giving consent. If a participant decides to withdraw, it will be clarified that they are only discontinuing the study treatment while continuing follow-up visits. Should a participant wish to fully withdraw from the study, their decision will be respected. Efforts will be made to determine the reason for withdrawal, although participants are not required to provide any explanation. Data already collected will be retained and included in the final analysis. The PI may also withdraw participants for various reasons, including but not limited to protocol violations, intercurrent illness, adverse events, serious adverse events, suspected unexpected serious adverse reactions, administrative reasons, or if participation in the trial interferes with ongoing care or results in symptomatic worsening.

In these cases, participants will continue to be followed up with the same schedule of research assessments as those who remain in the study, until they complete the 12-week follow-up period or until they develop frank psychosis, whichever occurs first. Participants who experience progression to first-episode psychosis will exit the study intervention, be classified as treatment failure, and only be assessed for safety outcomes until completing the 12-week follow-up period.

## 3. Discussion

Despite substantial advancements in early detection and prognosis, current research still converges towards the lack of effective interventions for transition to psychosis, subtle psychotic symptoms, functional status, and depression, among CHR individuals [3,5,56]. Noteworthy, APS, negative symptoms, and impairment in social and role functioning affect the quality of life of CHR individuals similarly to those of psychosis patients [57]. The present proof-of-concept Phase-2 study proposes the use of a nutraceutical, the fatty acid amide PEA in its ultramicronized form, to treat APS and reduce psychic distress in CHR individuals. In parallel, um-PEA modulation of potential CHR state biomarkers across the inflammatory, endocannabinoid, and microbiome systems will be assessed.

This study protocol should be seen considering some strengths and limitations. First, our study provides a reasonably extended treatment for help-seeking young adults that may not benefit from psychotherapy techniques and are often concerned about the possible unpleasant side-effects of psychotropic medications. Also, the use of conventional anti-psychotics before formulating the diagnosis of a clear-cut psychotic disorder raises concerns about the potential risk of accelerating disease progression [58], as well as implications from forensic and ethical standpoints [59,60].

Second, the trial will be pursued with minimal risks for study participants, being PEA a well-established Food for Special Medical Purposes (FSMP) for prolonged therapy in several clinical conditions [52], nearly devoid of known or potential adverse effects [61]. Oral PEA has shown well-documented safety and tolerability in numerous clinical trials with over 1000 individuals with doses 300–1200 mg per day both in healthy and sick populations [53,54], with negligible acute and repeat dose toxicity [62]. Also, no invasive techniques will be implemented throughout the study.

Third, the feasibility of this proof-of-concept trial will justify its continuation as a larger Phase-3 study investigating PEA efficacy, tolerability, and acceptance in CHR subjects.

To this extent, randomization and blinding of future studies will be needed to help reduce possible biases. Also, being a single-site study, selected CHR individuals may differ to some extent from the population treated in normal life, making it difficult to interpret and generalize the results. Indeed, in line with the evidence that around 50% mental health disturbances start by late adolescence [4], when patients are referred to transition-age youth psychiatry services [63], future studies would probably benefit from the inclusion of underage participants (e.g., aged 15–18 years). Moreover, although CHR youths with APS are those most referred to mental health outpatient services [64], future studies should also consider including patients from the CHR-vulnerability group and the CHR-brief limited intermittent psychotic symptoms (BLIPS) group [1]. This approach could help capture potential variations in clinical responses across the CHR spectrum. Finally, more stringent systems for monitoring patient compliance with therapy (e.g., medication diaries, text messaging [65]) would enable more rigorous tracking of medication adherence by participants in future trials.

Net of the above considerations, an open-label single-arm design appears suitable for an early-phase trial in this specific clinical population [66] and may represent the initial ground to build a greater understanding of the possible correlations between behavior and biomarkers modulation as a response to PEA supplementation in CHR individuals.

## Figures and Tables

**Table 1:** Study flowchart.

Procedures	Screening Phase	Feasibility Phase			Extension Phase	
	Screening (Day-7)	Baseline (Day 0)	T1 (4 weeks ± 7 Days)	T2 (12 Weeks ± 14 Days)	T3 (24 Weeks ± 14 Days)	T4 (36 Weeks ± 14 Days)
Informed consent	X					
Medical history	X					
Physical examination	X	X		X		X
Electrocardiogram	X	X		X		X
Urinalysis	X			X		X
*AUDIT* [37], *FTND* [38], *SDS* [39], *CEQ* [40]	X					
*TFLB* [41]		X	X	X	X	
Pregnancy test	X	X	X	X	X	
Hematology, biochemistry	X			X		X
Concomitant medication	X	X	X	X	X	X
Exclusion criteria	X	X				
Intelligence quotient	X					
*UKU-SERS* [42]		X	X	X	X	X
*Um-PEA* dispensing		X	X	X	X	
Compliance assessment			X	X	X	X
*CAARMS* [1]	X	X	X	X	X	X
*HADS* [43], *GF* Social/Role scales [44]		X	X	X	X	X
Microbiome, inflammation, eCBome blood and stool assessments		X	X	X	X	X

*AUDIT*, Alcohol Use Disorders Identification Test; *FTND*, The Fagerstorm Test for Nicotine Dependence; *SDS*, Severity of Dependence Scale; *CEQ*, Cannabis Experience Questionnaire; *TFLB*, The Timeline Followback; *UKU-SERS*, UKU Side Effect Rating Scale; *Um-PEA*, Ultramicronized-Palmitoylethanolamide; *CAARMS*, The Comprehensive Assessment of At-Risk Mental State; *HADS*, Hospital Anxiety and Depression Scale; *GF*, Global Functioning.

## Data Availability

The trial has been registered at Clinicaltrials.gov with identification code NCT06037993 (accessed on 5 December 2024). The patient information sheet and consent form are openly available at https://osf.io/nrc5b (accessed on 5 December 2024). The study case report form is openly available at https://osf.io/2vayh (accessed on 5 December 2024).
